# Behavioral effects induced by organic insecticides can be exploited for a sustainable control of the Orange Spiny Whitefly *Aleurocanthus spiniferus*

**DOI:** 10.1038/s41598-020-72972-x

**Published:** 2020-09-25

**Authors:** Selma Mokrane, Giuseppe Cavallo, Francesco Tortorici, Elena Romero, Alberto Fereres, Khaled Djelouah, Vincenzo Verrastro, Daniele Cornara

**Affiliations:** 1 International Centre for Advanced Mediterranean Agronomic Studies - Institute of Bari (CIHEAM-Bari), Via Ceglie 9, 70010 Valenzano, BA Italy; 2 Dipartimento Di Scienze Agrarie, Forestali Ed Alimentari DISAFA, University of Torino, Largo Braccini 2, 10095 Grugliasco, TO Italy; 3 Consejo Superior de Investigaciones Científicas (CSIC), Instituto de Ciencias Agrarias (ICA), Calle Serrano 115dpdo, 28006 Madrid, Spain

**Keywords:** Entomology, Environmental impact

## Abstract

The Orange Spiny Whitefly (OSW) *Aleurocanthus spiniferus* (Hemiptera: Aleyrodidae) represents a new serious threat to *Citrus* spp., grapevine and ornamental plants in the whole Mediterranean area. Such threat urgently calls for the development of a sustainable control strategy, including insecticides compatible with biological control, and applicable also in organic citrus farming that represent an essential part of Mediterranean agricultural economy. Therefore, we evaluated the toxicity and the effects on host searching, oviposition, and probing and feeding behavior exerted on OSW by organic insecticides supposed to have limited side effects on environment and ecosystem services, i.e. sweet orange essential oil (EO), extract of *Clitoria ternatea* (CT), mineral oil, pyrethrin and azadirachtin. Despite none of the compounds caused a significant mortality of any of the OSW instars, we observed interesting effects on whitefly behavior: (i) EO and pyrethrin showed a relevant repellent effect, with impairment of both adults landing and oviposition on treated plants; (ii) CT and pyrethrin strongly affected probing behavior. Here, in the light of our findings, we discuss possible OSW sustainable control strategies and further research perspectives.

## Introduction

Biological invasions are a pervasive component of global change, challenging the conservation of biodiversity and natural resources^1–5^. Many introduced populations remain innocuous for extended periods before spreading and becoming invasive^6^; by the time impacts are noted, irreversible ecosystem changes might have occurred^1,7^. Species introduced into new environments with accidental or intentional human assistance represent a serious threat for native species, ecosystems and human well-being^8–12^. A good example is represented by invasive arthropod crop pests for which control farmers often overuse insecticides^13–15^ with consequent side effects on non-target organisms^16^ and human health^17^.

Among invasive insect pests, many whiteflies (Hemiptera: Aleyrodidae) species have become widely distributed due to anthropogenic activities, especially national and international trade, with dramatic consequences for agriculture, due to their ability to cause direct damages to the crop and to transmit plant viruses^18,19^. Due to habitat fragmentation creating abundant and diverse niches, Italy is one of the most welcoming territories in Europe for foreign species, such as the Orange Spiny Whitefly (OSW) *Aleurocanthus spiniferus* (Quaintance, 1903) (Hemiptera: Aleyrodidae). OSW, a whitefly species originating from China and South and Southeast Asia, was reported for the first time in 2008 in the EU territory in Southern Italy, Apulia Region^20^. Currently, OSW has been found in central and northern regions of Italy, beside other countries across the European Adriatic coast as Croatia, Montenegro and Greece^21–26^. OSW is currently included in the EPPO list (A2) as a quarantine pest threatening Europe. The main OSW host plants are *Citrus* spp.: infested plants show a general weakening due to sap loss, and a severe reduction of photosynthesis and respiration activities caused by sooty mold developing on the abundant honeydew produced by the whitefly that completely covers the plant. Eventually, heavily infested trees have an almost completely black appearance, and the fruits are unmarketable^24^. Infestations of *Citrus* spp. plants by the sibling species *Aleurocanthus woglumi* (Ashby, 1915) (Hemiptera: Aleyrodidae) have been reported to reduce fruit setting by ca. 80%^24^. In addition, the whitefly seems rapidly adapting to the European environment; indeed *A. spiniferus* is creating new trophic associations with previously unreported host plants, such as grapevine, *Parthenocissus* sp., *Ailanthus altissima*, and other cultivated and ornamental plants^21,24,26^. However, such plants can be just transient hosts serving as shelter during periods when the main hosts, i.e. *Citrus* spp. plants, become unsuitable/less suitable^24,26^. Beside the economic losses, the spread of OSW in Italy is considered as having serious environmental consequences. Indeed, the growers reacted to infestations with a massive use of synthetic wide-spectrum insecticides applied untimely and without technical criteria that, besides having proved ineffective, might have caused serious side effects on the environment and non-target arthropods, together with the possible development of resistant whitefly populations^21,24,26–28^. As observed by Vieira et al*.*^28^ for the congeneric species *A. woglumi* in Brazil, the use of mineral oils, vegetable oils, or derivate, may result in improved control strategies for the pest with minimal adverse effects on populations of natural enemies and non-target species^28^. Indeed, low-impact organic certified pesticides could be integrated with augmentative biological control^21,26,29^, developing a sustainable strategy for the management of OSW. Such strategy, important in conventional agriculture to produce safe and sustainable products, would be crucial in organic farming, where relatively few pesticides are available and allowed (EU Regulation (EC) No 889/2008)^30^. Furthermore, pesticides should be evaluated not just depending on short-term efficacy, but looking at direct and indirect effects possibly altering pest physiology and behavior, with consequences irradiating from the single individual exposed to the toxicant, to population and community-level^16,31^. Overall, the implementation of a sustainable and effective OSW control strategy applicable in organic *Citrus* spp. farming, which represents an important part of the agricultural economy of the entire Mediterranean area^32^, is an urgent issue of paramount importance. Therefore, we tested the effects of different organic-certified pesticides on OSW, both in terms of lethal toxicity and of sublethal effects on insect’s behavior, i.e. on host searching, landing, oviposition, and probing and feeding behaviors.

## Results

### Lethal toxicity for the different stages of *Aleurocanthus spiniferus*

**Figure 1:**
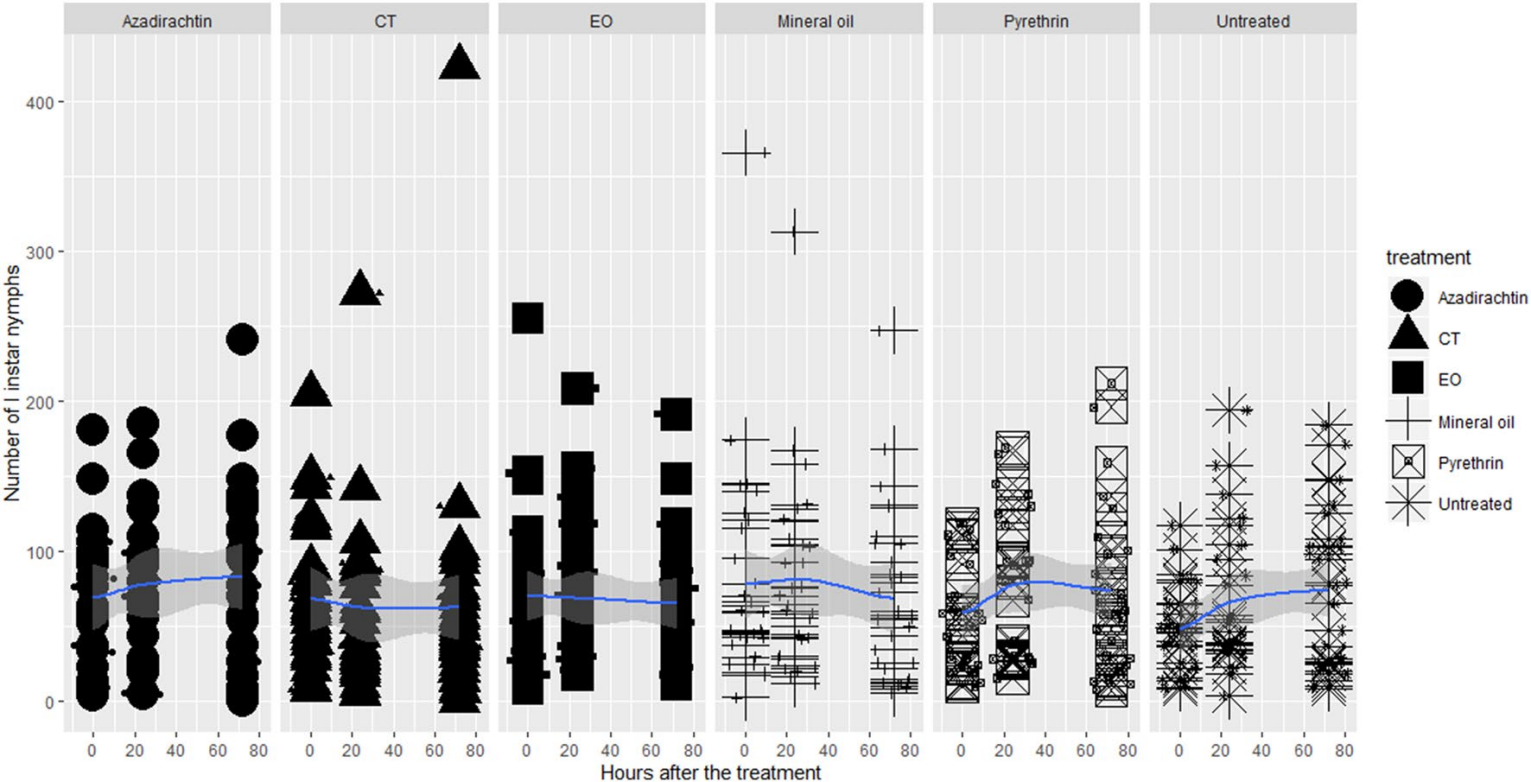
Lethal toxicity of the insecticides on OSW I instar nymphs.

**Figure 2:**
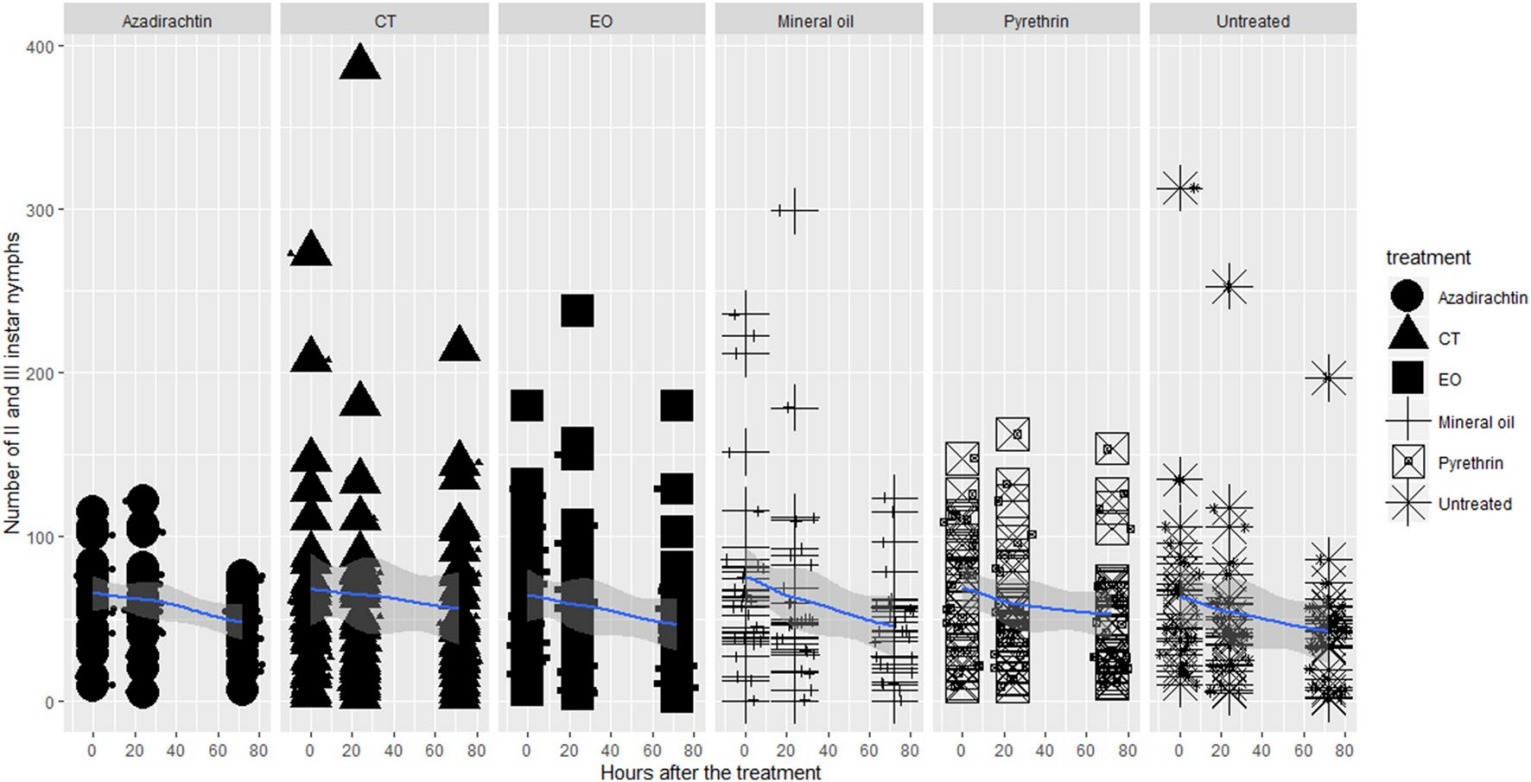
Lethal toxicity of the insecticides on OSW II and III instar nymphs.

**Figure 3:**
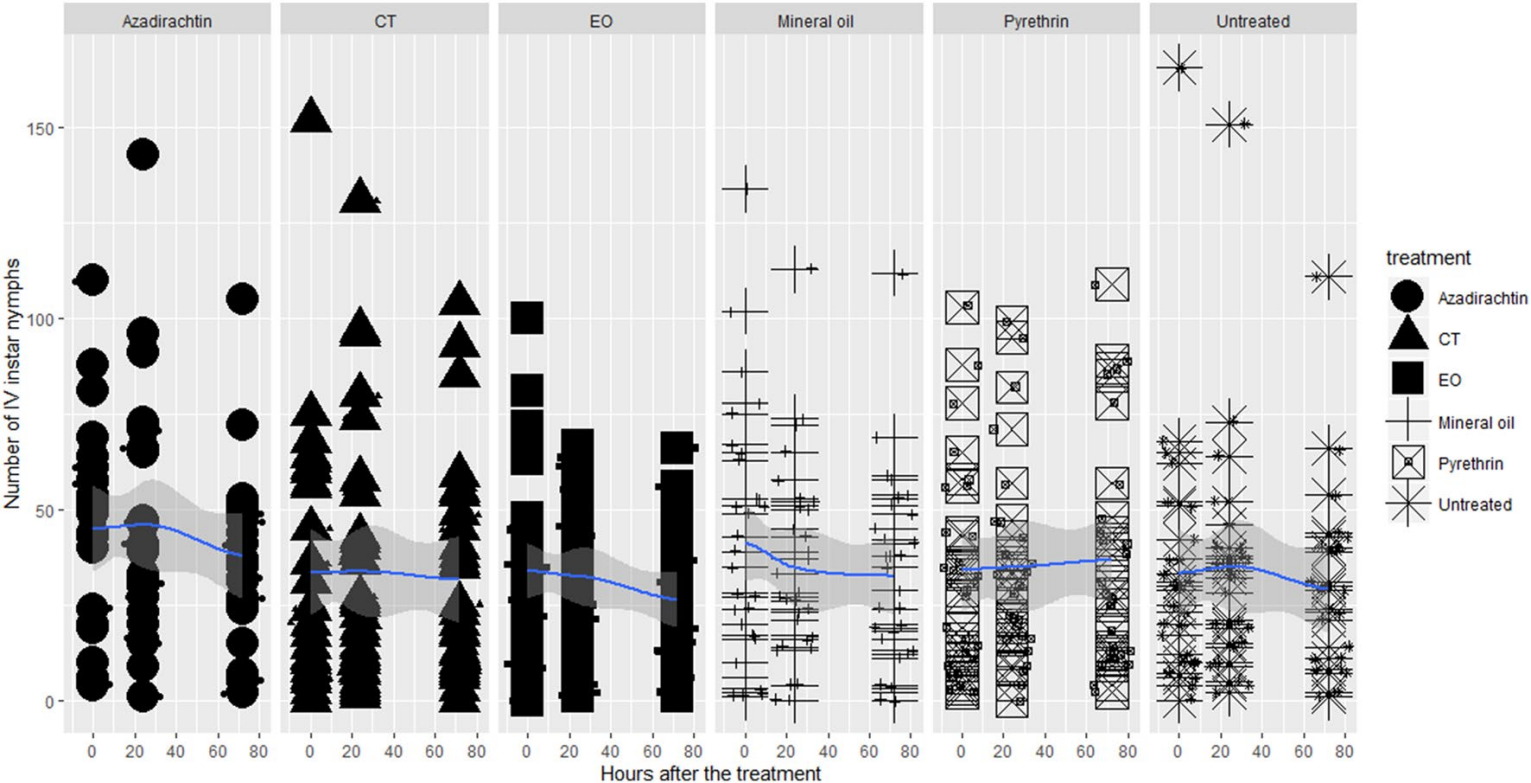
Lethal toxicity of the insecticides on OSW IV instar nymphs (puparia).

**Figure 4:**
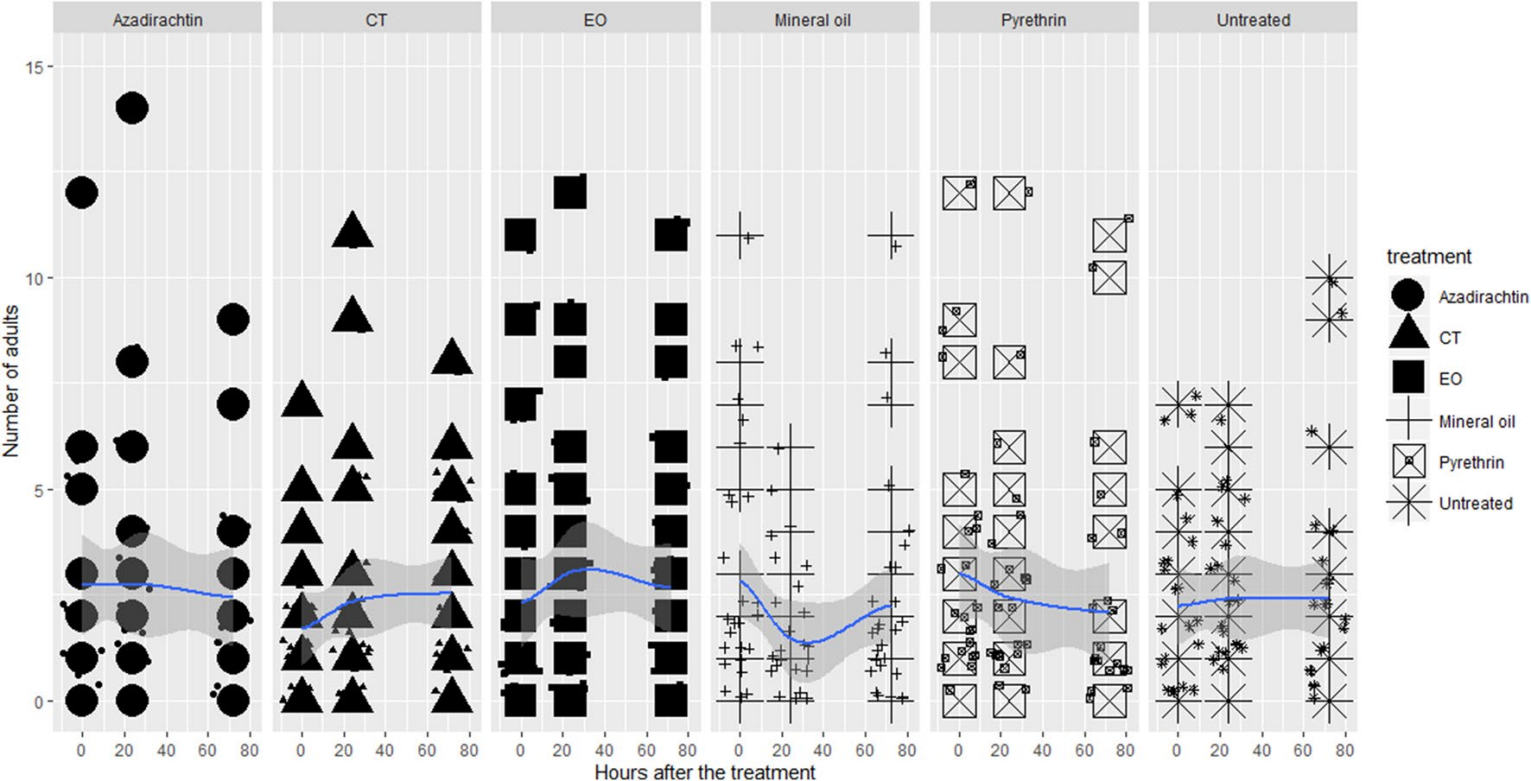
Lethal toxicity of the insecticides on OSW adults.

The products caused no statistically significant mortality in any of the OSW pre-imaginal instars, with no differences among the different insecticides according to the results of the Tukey tests (Table S1; Figs. 1, 2, 3 and 4).

Regarding OSW adults, the effect of treatment was significant for CT (z = − 3.686, *p* < 0.001), EO (z = − 2.6, *p* = 0.009), mineral oil (z = − 2.910, *p* = 0.003), pyrethrin (z = − 2.059, *p* = 0.039) and control (z = − 2.696, *p* = 0.007). Considering the pairwise comparison (Tukey test), plants sprayed with azadirachtin showed significantly more adults than other treatments including control (Table S1).

Adults’ emergence from puparia was not affected by the tested compounds.

**Figure 5:**
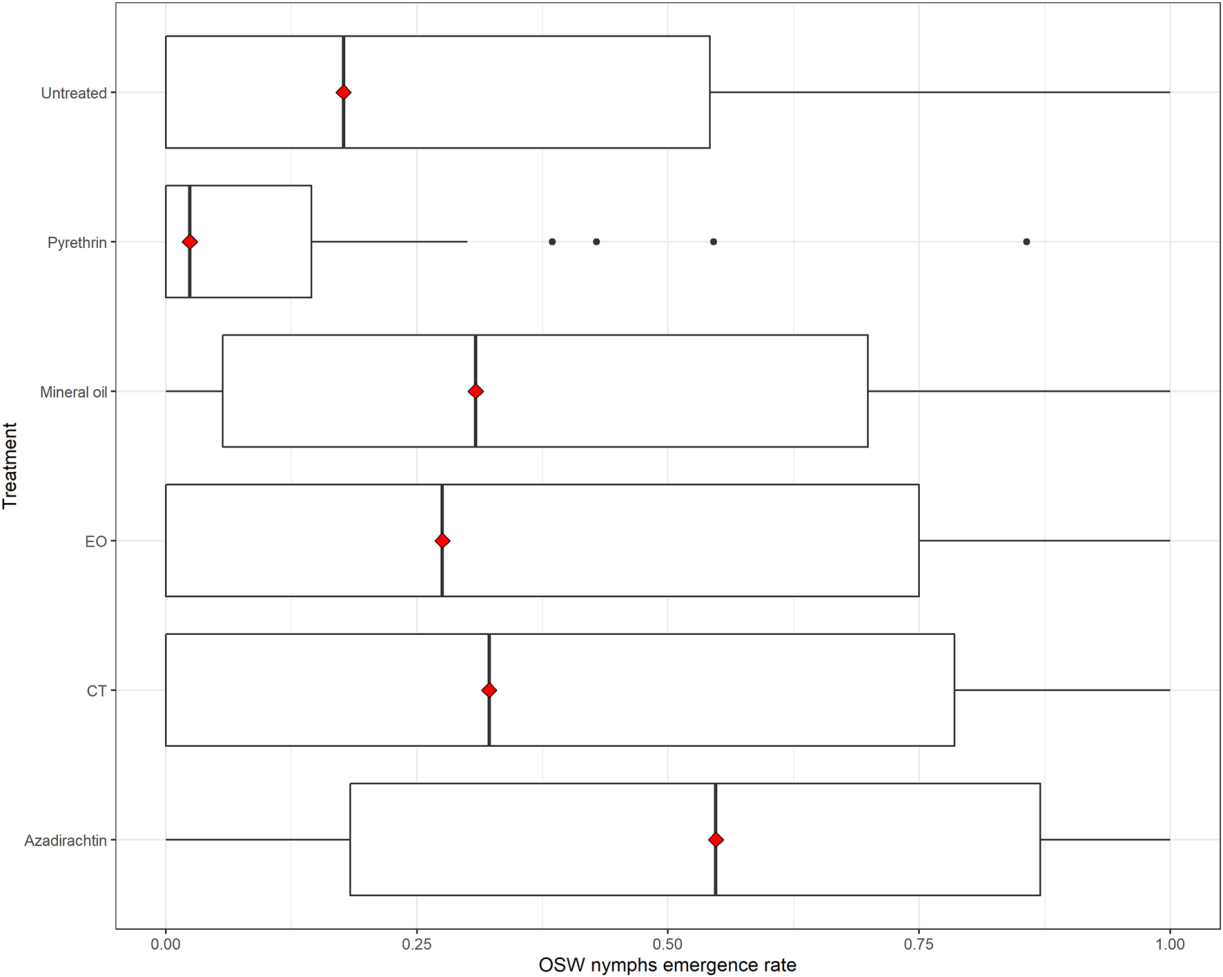
Effect of the insecticides on eggs hatching, i.e. number of first instar nymphs emerged/alive upon emergence over the total number of eggs laid. Red squares indicate median values.

Treatments significantly differed considering the number of nymphs emerged (and alive) from sprayed eggs (Kruskal–Wallis chi-squared = 30.474, *p* < 0.001) (Fig. 5). Pyrethrin caused the greatest reduction in the number of emerged and alive first instar nymphs compared to the other substances tested (Table S2 and S3). Pyrethrin difference with the control was just close to the statistical significance (*p* = 0.07); number of alive nymphs for the control was marginally significantly lower than azadirachtin (*p* = 0.048), and similar to CT (*p* = 0.40), EO (*p* = 0.67), and mineral oil (*p* = 0.40) (Table S3).

### Sub-lethal effects on *Aleurocanthus spiniferus* host searching and oviposition behavior

**Figure 6:**
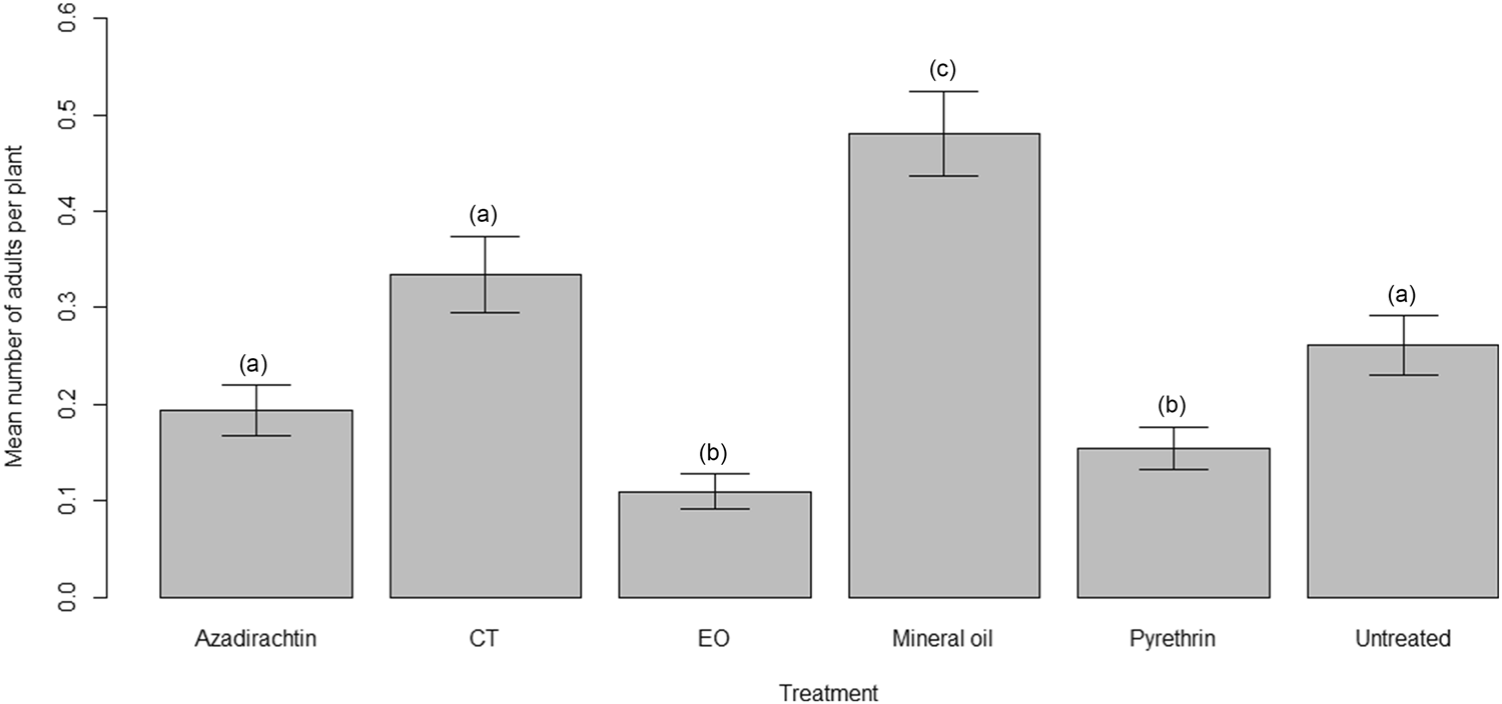
Effect of the insecticides on OSW adults host searching behavior, i.e. on the number of adults alighting on citrus plants. Letters within brackets indicate significant differences according to pairwise comparison.

The organic compounds tested had significant effects on both the host searching/alighting behavior and the oviposition. We observed a significant difference in the number of adults alighting on the citrus plants among the treatments (Kruskal–Wallis chi-squared = 83.879, *p* < 0.001). Considering the pairwise comparisons, the greatest number of adults was observed for plants sprayed with mineral oil that significantly differed from the other treatments. Values for azadirachtin, CT and control were overall similar. Plants treated with EO and pyrethrin were the ones showing the lowest number of adults, with no difference between them (Table S5 for the pairwise comparison using Wilcoxon rank sum test and Fig. 6).

**Figure 7:**
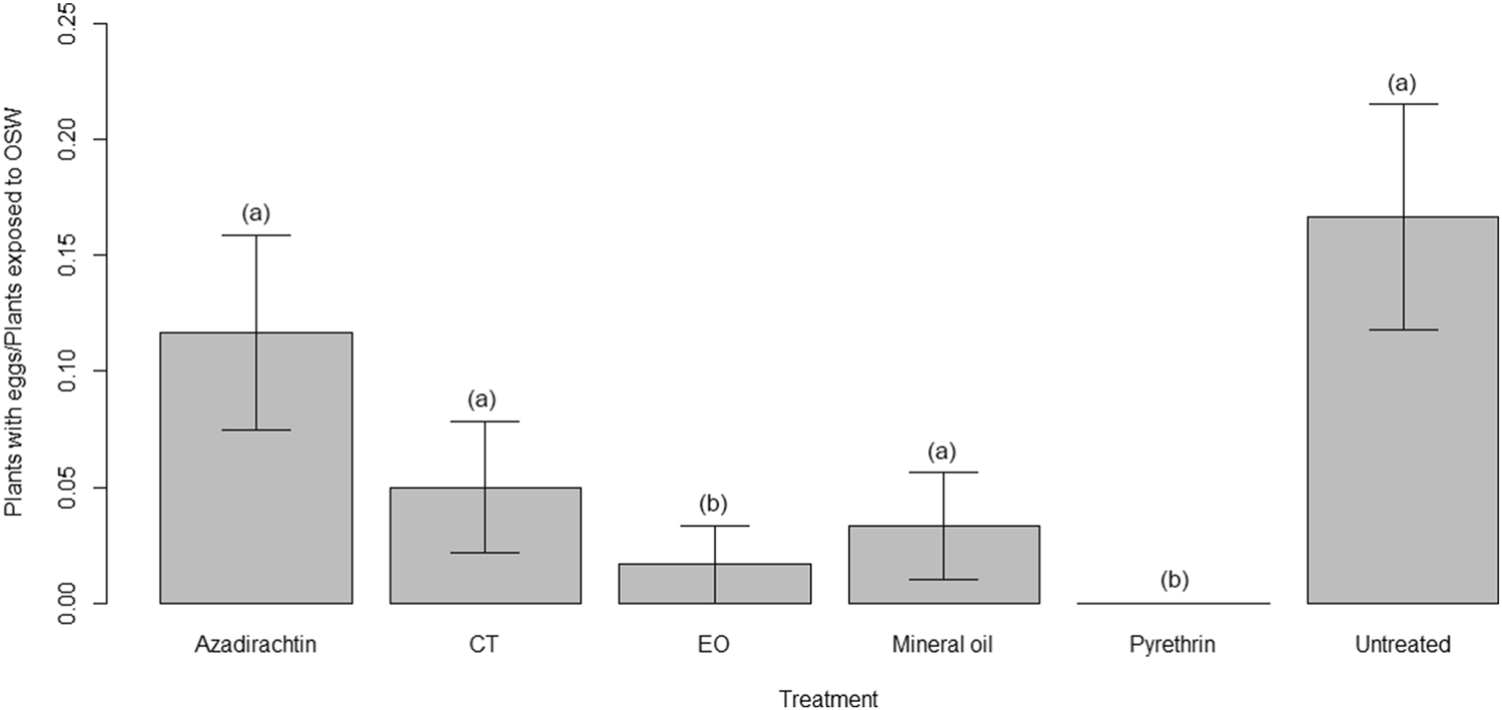
Effect of the insecticides on OSW oviposition, i.e. on the number of plants on which OSW laid eggs. Letters within brackets indicate significant differences according to pairwise comparison.

Treatments significantly differed also for their effect on oviposition, i.e. the total number of citrus plants on which OSW laid eggs (Kruskal–Wallis chi-squared = 20.796, *p* < 0.001). The greatest number of plants with eggs was recorded for the untreated control, that did not differ from azadirachtin (*p* = 0.504) and CT (*p* = 0.103); the difference with mineral oil was just marginally non-significant (*p* = 0.058). Oviposition was instead significantly impaired on plants sprayed with EO and pyrethrin (on the latter no oviposition occurred), with no difference among the two treatments (Table S6 for the pairwise comparison using Wilcoxon rank sum test and Fig. 7).

### Electrical Penetration Graph-assisted evaluation of the *Aleurocanthus spiniferus* probing and feeding behavior

Regarding the Electrical Penetration Graph (EPG) results, overall just few adults performed behaviors related to phloem and xylem activities (G NWEI: 2 adults in untreated, 2 in EO, 1 in pyrethrin, 0 in CT; E1 NWEI: 1 in untreated, 1 in EO, none in pyrethrin and CT; E2 NWEI: 1 in untreated, 1 in EO, none in pyrethrin and CT). Furthermore none of the monitored adults performed E1e and pd. OSW on plants treated with pyrethrin showed significantly lower number of probes than CT (z = 2.720, *p* = 0.033), EO (z = 3.199, *p* = 0.007), and untreated (z = − 2.972, *p* = 0.015). We observed no significant difference among the treatments in np WDI. On the contrary, OSW on pyrethrin-treated plants showed significantly shorter C waveforms (C WDI) than EO (z = 3.006, *p* = 0.014) and untreated (z = − 3.052, *p* = 0.012), significantly shorter Total Probing Time than EO (z = 2.836, *p* = 0.023) and untreated (z = − 2.937, *p* = 0.017), and significantly longer time needed for the 1^st^ probe than EO (z = − 3.424, *p* = 0.003) and untreated (z = 3.371, *p* = 0.004). There was no significant difference between pyrethrin and CT regarding the latter three EPG’s variables.

## Discussion

Pest control could be achieved through the integration of multiple tactics; among the possible alternatives to synthetic pesticides, the use of chemical stimuli to manipulate insect behavior is considered a suitable biotechnical control tool in the management of several insect pests^29^. Some insecticides, beside lethal toxicity, affect insect behavior preventing settling on the host plant, probing and feeding, oviposition, and virus transmission^33,34^. Therefore, behavioral effects caused by an insecticide that is apparently non-toxic for the insect in conventional tests, could in fact permit to contain the insect populations and associated yield losses. As remarked in the introduction to this paper, massive untimed and mainly unjustified use of synthetic pesticides following the introduction of OSW in Italy was ineffective, beside possibly causing the emergence of resistant whitefly populations^21,24,26–28^. The current relentless spread of OSW over the Mediterranean together with the threat posed to *Citrus* spp., grapevine and ornamental plants, call for the urgent search of alternatives to conventional control strategies. In this perspective, botanicals and in general organic-certified insecticides could represent a sustainable tool. However, recent works highlighting previously overlooked biopesticides’ lethal and sub-lethal effects on natural enemies^35,36^, warn for a reconsideration of the general concept of their safety for non-target organisms, and for more accurate risk-assessments^37–41^. Organic-certified pesticides could be even integrated with synthetic insecticides in alternate treatment regimes, preventing or at least delaying the development of resistance, and reducing the environmental impact of pest control practices^37,42^. However, as stated by Copping and Menn^37^ referring to botanicals, there is a general low level of acceptance of organic-certified pesticides given their limited shelf-life, and inconsistent results. Nonetheless, considering inconsistent results, most of the trials performed with organic pesticides (and pesticides in general) focus on lethal toxicity rather than considering also the sub-lethal effects, i.e. physiological and behavioral effects caused by the insecticide on the pest survived to exposure^16,41^. Indeed, in contrast with previous technical reports^43^, the pesticides tested in our experiments caused no significant mortality in any of the OSW instars, although interesting sub-lethal effects that could be exploited in an environmentally sustainable IPM strategy were observed. In this study, to have a complete picture of the effects of the organic insecticides under screening on OSW, we used a complex approach integrating classical field mortality trial, laboratory free-choice and oviposition experiments, and EPG. Several authors have used EPG to assess the effect of insecticides on insect probing and feeding behavior^44–47^. In our case, EPG furnished important indications on OSW reaction to the different compounds. However, lack of phloem activities in our recordings might suggest OSW females were strongly affected by the tethering technique, different from the one indicated by Chesnais and Mauck^48^ as the most suitable for the whiteflies. An alternative and intriguing explanation to the observed phenomenon, that deserves to be investigated in the future using different host plants and comparing nymphs and adults, is that adults’ feeding activity at least on sweet orange is limited, thus this plant is used by adults as mating and oviposition site rather than food source. Still, we consider EPG data collected in this experiment as reliable and useful for our evaluation, since our purpose was limited to a treatments comparison.

Mineral oil influenced the host searching behavior, with more OSW adults observed landing on plants treated with these compounds compared to other treatments and control. Even though more adults landed on mineral oil-sprayed plants, the total number of plants where oviposition occurred was lower than other treatments and control. However, impairment of oviposition was statistically significant just for EO and pyrethrin (the latter compound entirely impaired oviposition, with no plants with eggs).

CT (the extract of *Clitoria ternatea*) significantly affected OSW probing behavior, reducing the time spent by the whitefly in probing activities. We decided to include in the EPG design only CT excluding mineral oil, in order to simplify the experimental design, and given CT has been proposed as alternative for the gradual phase-out of mineral oil in organic agriculture., Indeed, given their nature and their controversial and still unclear effects on environment and non-target organisms, mineral oils should be considered as contentious input particularly in organic agriculture, and their at least partial replacement with other substances is desirable^49–52^. During the experiments, we noticed that upon the treated plants, CT created a surfactant layer coating the tissues, similarly to mineral oil. This layer could have impaired OSW host-searching/recognition, namely those actions/behaviors performed by the insect to recognize the host-plant, including probing^53^. At the same time, the negative impact on feeding could have been related to the high concentration of cyclotides presents in *C. ternatea* extract, that were demonstrated to affect larval growth of *Helicoverpa armigera* (Lepidoptera: Noctuidae) in a dose-dependent manner when administered through diet^54^. Therefore, timely application of CT could impair probing and feeding and settling, overall reducing OSW population levels. Sweet orange essential oil (EO) did not show the insecticidal effect on OSW reported by Guario et al*.*^43^; however, the reduced number of adults landing on the treated plants and the impairment of oviposition, possibly associated to the synergistic action of limonene with other compounds, are consistent with the EO repellent effect reported for other insects^34,55–58^. Nonetheless, EO had no effect on the probing behavior that did not diverge from that displayed by the whitefly on untreated plants. Repellency without immediate significant effects on probing behavior had been observed also by Powell et al*.*^44^ for polygodial on aphids; however, the same authors reported changes in probing behavior following longer exposure to polygodial, a sub-lethal effect that deserve to be invested for EO too, given previous observations on essential oils inhibitory activity of acetylcholinesterase^34,59^.

Pyrethrin resulted to be the most promising organic-certified compound for the control of OSW. Pyrethrin was highly effective in reducing nymphs’ emergence from eggs when plants with eggs were treated with the compounds under screening, although no significant first instar nymphs mortality was observed in field. Consistently with Campolo et al*.*^55^, we believe the observed phenomenon is not the result of an effect of pyrethrin on eggs, since the structure of the eggs protects the developing embryos and may interfere with insecticide penetration^60^. Instead, pyrethrin could have directly acted on the nymphs upon emergence, through either lethal toxicity (not consistent with our field result) or feeding-deterrent effect (consistent with our EPG data), similarly to what we observed with adults. Indeed, according to our EPG results, pyrethrin strongly repelled adults, that barely attempted to probe the plant and, when occurring, for a very short time. In our test with pyrethrin treatment on plants with eggs, the plants were stored upon treatment inside a growth chamber, thus with no direct exposure to sun light. This could have exponentially increased pyrethrin persistency that is limited under field conditions due to compound’s photo-instability^37^. Persistency issue could be fixed with pyrethrin’s micro- or nano-encapsulation, with an overall reduction of oxidation, hydrolysis and other degradations^55,61^. Finally, pyrethrin completely impaired oviposition, with no eggs laid by the adults on treated plants.

Overall, our results highlight how pyrethrin (especially micro- and nano-encapsulated formulations), CT, and EO, if timely applied, could be successfully employed for OSW integrated control strategies. Indeed, even if induced mortality seems not particularly relevant, they could interfere with pest’s perception of plant cues necessary for host selection, settling and feeding^62^. However, it is worth mentioning that our results on OSW mortality rate refer to single exposures of the insects to the products tested; therefore, we consider important to assess the efficacy of repeated treatments with the tested products in a relatively short time-span, through field trials that might furnish essential technical indications to farmers and stake-holders. Additionally, results we obtained in free-choice tests could have been influenced by the presence within the same cage of all the products (each plant was treated with one product), thus with a co-existence of different scents that could have affected OSW adults orientation. Although our design is closer to a field situation where different scents co-exist within the same environment, final evidences on repellent/attractive properties of each compound alone should be assessed with further experiments.

The main advantage of the organic compounds we tested, at least theoretically (as discussed above), is their relative safety for non-target-organisms compared to conventional pesticides, thus the possibility of using them in combination with biological control, the latter proven to be effective against OSW^21,26,63^. Regarding biological control, following OSW first detection in Europe, efforts were put in place to find suitable candidates for the whitefly biological control^21,26^. In parallel to our experiments, during August 2019, 80 OSW-infested leaves of *Citrus* spp. (lemon, orange, tangerine) surrounding the CIHEAM Bari experimental field were randomly collected, isolated singly in Petri dishes covered with aphid net and sealed with rubber band, and stored in a growth chamber (24 ± 2 °C, 60%RH, 14:8 L:D) for two weeks. Two parasitoid wasp species emerged from the samples, *Eretmocerus mundus* Mercet (Hymenoptera: Aphelinidae) (a total 62 individuals emerged from 13 leaves) and *Telenomus* sp. (Hymenoptera: Scelionidae) (a total of 24 individuals emerged from 19 leaves), together with OSW adults. It was not possible to associate the parasitoids with specific whitefly instars. *Eretmocerus* spp. were reported as parasitoids of *A. spiniferus* in the South-East Asia^21,64^. In addition, *E. mundus* is an effective parasitoid of the whitefly *Bemisia tabaci*, with a preference for second and third instar nymphs^65,66^. The association of *Telenomus* with Aleyrodidae has never been recorded, and the present work cannot confirm any possible association. *Telenomus* host range include a large amount of genera of different Orders; another genera of the superfamily Platygastroidea, *Amitus* Haldeman (Hymenoptera: Platygastridae), was recorded as parasitoids of *Aleurocanthus* spp.^64,67^, Further studies on these two parasitoids exploring their possible relationship with OSW are urgently needed.

The development of a sustainable integrated pest management strategy, and the recourse to novel environmentally-friendly control tactics, as for example manipulation of vibrational communication^68,69^, requires a clear understanding of pest biology and behavioral/chemical ecology of its interaction with host plants, conspecifics, natural enemies and microorganisms^29^; unfortunately, such knowledge on OSW in the Mediterranean agro-ecosystem is missing. Therefore, containing the threat posed by this insect to Mediterranean agriculture will require massive research efforts focused on: (1) filling the knowledge-gap on fundamental elements of the whitefly bionomics and landscape ecology; (2) searching for natural enemies to employ in conservation and augmentation programs, testing their efficacy in containing OSW population together with their susceptibility to the pesticides to be used in integrated control strategies.

## Methods

### Tested insecticides

The products tested in our experiments were: (i) sweet orange (*Citrus sinensis* L.) essential oil (herein referred as EO); (ii) an extract of *Clitoria ternatea* (herein referred as CT); (iii) mineral oil; (iv) pyrethrin; (v) azadirachtin (see Table 1 for more details). Choice of these organic-pesticides was driven by previous reports of their efficacy against OSW, as in the case of EO^43^, or effectiveness on other whiteflies, as for CT against *Trialeurodes vaporariorum*^70^, or their widespread use in organic agriculture, i.e. pyrethrin, azadirachtin and mineral oil (EU Regulation (EC) No 889/2008). For the tests we applied the maximum doses indicated in the products’ label. Plants treated with tap water were used as control.

**Table 1:** Organic-certified insecticides tested against OSW.

Active ingredient	Acronym	Commercial name	Company	Dose ml/L^a^
Sweet Orange Essential oil	EO	PREV-AM	Oro Agri, Inc	6
Extract of *Clitoria ternatea*	CT	Sero-X	Bi-PA—Biological Products for Agriculture Technologielaan	20
Mineral oil	Mineral oil	Ufo	Biogard	20
Azadiractin	Azadiractin	Oikos	Sipcam	1.5
Pyrethrin	Pyrethrin	Pyganic 1.4	Biogard	1.5

^a^Treatments were carried out with label doses.

### Lethal toxicity for the different stages of *Aleurocanthus spiniferus*

#### Field trial

Firstly, we evaluated the efficacy in terms of induced mortality of the five pesticides on OSW different instars in an experimental field located in the premises of the CIHEAM-Bari research institute (Apulia region, Southern Italy). The experimental field was composed by 84 two-year old sweet orange (*Citrus sinensis*) plants var. Valencia late, grafted on sour orange (*Citrus aurantium*) rootstock, planted on eight rows of ten plants each, plus a central row with four plants. At the moment of the trial, the plants had a height of approximately 1 m. The plants were fertilized monthly (Anticlor FE24, l.gobbi) and watered three times per week. The plants were naturally infested with OSW possibly dispersing from heavily infested lemon (*Citrus limon*), orange, and tangerine (*Citrus reticulata*) plants located ca. 30 m far from the experimental field (on the Eastern side). OSW fourth instar nymphs (pupal cases) were collected on the orange plants of the experimental field, prepared and slide-mounted according to Pizza and Porcelli^71^ protocol, and identified at species level according to the taxonomical keys of Martin^72^ and Jansen and Porcelli^19^.

The insecticide treatments were carried out during the last week of July (field conditions: temperature 26 ± 6 °C; RH 61 ± 13%) and the second week of September (field conditions: 28.3 ± 5 °C; 58.8 + -14.5% RH) (thus, two separate replicates). During each date, 16 plants were sprayed with EO, 15 each with CT and the mineral oil, 12 with azadirachtin, and 13 each with pyrethrin and tap-water (the latter used as control). The sprayed plants were distributed following a completely randomized block design. The products were sprayed with a low-pressure knapsack sprayer (Ausonia 38018, 16L) until runoff.

Presence and abundance of the different OSW instars on each plant were assessed at three moments (surveys): (i) the day before the treatments; (ii) 24 h after the treatments; (iii) 72 h (3 days) after the treatments. During each survey, ten leaves per plant were scrutinized in field with a × 10 magnifying lens, in order to evaluate: (i) number of I instar nymphs; (ii) number of II and III instars nymphs; (iii) number of IV instar nymphs; (iv) number of adults. The ten randomly selected citrus leaves per plant were marked with a tape during the first survey (24 h before the treatment); the same leaves were scrutinized during all the surveys (repeated measures).

#### Effects on adults’ emergence from puparia

Additionally, following the last field survey (72 h after the treatment), two randomly-chosen leaves per plant carrying OSW puparia (IV instar) were collected, placed in aerated Petri dishes covered with aphid-net, sealed with rubber band, and stored in a climatic chamber (27 °C, 70%RH, 16:8 L:D photoperiod). Adults emergence rate was checked daily for two weeks until no adults were found on the Petri dishes.

#### Effects on eggs hatching

Finally, we assessed the effect of the tested compounds on eggs hatching, i.e. on the number of nymphs emerged/alive over the total number of eggs laid by OSW females before the treatment. Briefly, 60 one-year old sweet orange plants var. Madame Vinous were caged inside three cages (20 plants/cage) containing each 100 OSW adults reared under controlled conditions (males and females, ratio = 1:1) (whitefly rearing described below). The cages (Bug Dorm 2120) containing the plants and the adults were kept inside a growth chamber under controlled conditions (24 ± 2 °C, 60%RH, 14:8 L:D). Three days after the exposure to adult whiteflies, the plants were checked for presence and number of eggs by using an × 10 magnifying lens. Plants with eggs were treated with the tested compounds (and water for untreated control) with a hand-handle sprayer (Matabi IK, 1.5L) until runoff. The different treatments were kept separated, confined inside rectangular cages (1 × 1 m, metal frame, covered with aphid-net), one cage per treatment, and maintained in a growth chamber under controlled conditions (24 ± 2 °C, 60%RH, 14:10 L:D). The plants were daily screened for the emergence of the first instar nymphs for ten consecutive days. The experiment was replicated three times. For obtaining adults OSW to use in this trial, two year-old field infested grapevine potted plants var. Cabernet Sauvignon were caged inside Bug Dorm 2120 , six plants per cage. The cages were kept into a climatic chamber under controlled conditions (24 ± 2 °C, 60%RH, 14:10 L:D), and water-fertilized three times per week (Anticlor FE24, l.gobbi). Two to three day-old adults emerging from the puparia on the grapevine plants were used for the experiment (and for the experiments described below).

#### Sublethal effects on *Aleurocanthus spiniferus* host searching and oviposition behaviors

We evaluated whether the compounds under screening affected OSW host searching behavior, together with settling and oviposition. Two to three-day-old adults emerging from the puparia on the grapevine plants (method described above for the trial on eggs) were used for the experiment. Twenty-four two-year old sweet orange var. Madame Vinous plants per replicate having the same vegetative conditions (40 cm height, ca. 12 leaves per plant) were treated with the six compounds under screening. Thereafter, the plants were caged inside four Bug Dorm cages, six plants per cage, each plant corresponding to a treatment. The cage was ideally divided into six squares (each square corresponds to a position), namely A, B, C, D, E, F (Table S4). We took note of the position of the plant inside the cage, thus of the square occupied by the plant/treatment; in each cage, we switched the position of each treatment, thus the square occupied by the treatment, in order to avoid position effects. Three hours after the treatment we introduced in each cage ten OSW adults (ratio male:female = 1:1). We took note of the position of the OSW adults inside the cage, i.e. the number of adults on each plant/treatment, one, three, 14, 24, 48 and 72 h upon the introduction. Following the last survey (72 h), the plants were checked for oviposition (number of plants with eggs). We carried out 15 replicates, thus a total of 60 plants per treatment.

#### EPG-assisted evaluation of the *Aleurocanthus spiniferus* probing and feeding behaviors

We evaluated the possible effect of CT, EO and pyrethrin on OSW probing and feeding behavior by EPG (Electrical Penetration Graph)^73^, a tool that permits to characterize in detail the interaction of insects with sucking-probing mouthparts with plants and plant pathogens^74,75^, often applied also in studies on insecticides^76^. We choose these three compounds according to the results gathered by the experiments described above (see discussion section for further details). The insects used for this trial were two to three day-old adults obtained from field infested grapevine plants (method explained above for trial on eggs). Upon collection, the adults were moved for 24 h to other BugDorms cages containing each six two-year old sweet orange plants var. Madame Vinous potted in 1L pots filled with garden soil, peat, and pumice, for acclimation before the EPG. The cages were kept into a climatic chamber under controlled conditions (27 °C, 70%HR, 16:8 L:D). For the EPG, females OSW were tethered according to the protocol by Moreno-Delafuente et al*.*^77^. Upon tethering, each adult was placed on the abaxial surface of an apical leaf of a sweet orange plant treated with one of the substances under screening; the plants were sprayed with a hand-handle sprayer until runoff three hours before the EPG recording, with the same doses used for the other experiments (Table 1). The plants used for the EPG were two-year old sweet oranges var. Madame Vinous (the same as the plants used for OSW rearing). We recorded the behavior of four adults OSW per time (thus four adults per recording), with each whitefly placed on a plant to which one of the four treatments was applied. Therefore, each EPG recording was carried out with the four treatments simultaneously (four plants, each plant corresponding to one of the three treatments plus control). The position of the plants/treatments was switched during the different recordings in order to avoid position effects. We carried out ten replicates (recordings) for each treatment.

The recordings were carried out inside a Faraday cage, in an acclimatized room (23 ± 2 °C). Probing and feeding behavior was recorded for six hours with a Giga 8-DC EPG (EPG-systems, Wageningen, The Netherlands) at 1 Giga Ohm input resistance. Output from the EPG at 100 × gain was digitalized at a rate of 100 samples per sec. per channel, and recorded using Stylet+software (EPG-systems, Wageningen, The Netherlands; https://www.epgsystems.eu). Substrate voltage was adjusted following the calibration instructions of the DC EPG equipment so that EPG output signals fit into the + 5 V to − 5 V window provided by the software. EPG waveforms were interpreted according to Janssen et al*.*^78^ and Moreno-Delafuente et al*.*^77^. As variables needed to describe the effect of the treatments on OSW probing behavior we calculated: the total number of probes performed by the whitefly during the 6 h (n probes); the total duration of each of the waveforms np (non-probing), C (stylets pathway), G (xylem-feeding), pd (potential drop), E1e (extracellular salivation); E1 (phloem salivation); E2 (phloem ingestion) during the 6 h (WDI); the total number of each of the waveforms G, pd, E1e, E1, E2 performed during the 6 h (NWEI); the total time spent by OSW in probing activities during the 6 h (Total probing time); the time required by the whitefly to perform the first probe from the beginning of the rec (Time to the 1st probe).

### Statistical analyses

Statistical analyses was performed by using the software R 3.5.3 (R Core Team 2019)^79^. The efficacy of the products in terms of induced mortality on OSW different instars was assessed through Negative Binomial Generalized Linear model (glm.nb) (“MASS” package)^80^, with treatment, time, and their reciprocal interaction as explanatory variables. In case of significant difference among treatments, multiple comparisons with the Tukey test (Tukey’s post-hoc test) (*p* < 0.05) were performed using the “emmeans” package^81^, with adjusted *p* values. We checked the models for overdispersion and residual distribution using the “DHARMa” package^82^. The effect of the compounds on adults’ emergence from puparia, eggs hatching , host searching behavior (number of adults on the plants), and oviposition (number of plants with eggs per treatment) was assessed by Kruskal–Wallis rank sum test, followed in case of significant differences by pairwise comparisons using Wilcoxon rank sum test.

Treatments effect on OSW probing behavior (i.e. on the EPG’s variables) was evaluated through generalized linear model (GLM) with quasiPoisson distribution to compensate for overdispersion and Tukey test for pairwise comparisons.

## Supplementary information

Supplementary information.

## Data Availability

Results from statistical analysis are provided in Supplementary Materials. Additional data will be furnished by the authors upon reasonable request.
