# Supplementary material for: Behavioral effects induced by organic insecticides can be exploited for a sustainable control of the Orange Spiny Whitefly Aleurocanthus spiniferus
Source: Sci Rep. 2020 Sep 25;10:15746. doi: 10.1038/s41598-020-72972-x (PMC7519102; doi:10.1038/s41598-020-72972-x)
Supplement: Supplementary file 1 — Supplementary information. [file 41598_2020_72972_MOESM1_ESM.docx]

**SUPPLEMENTARY MATERIALS**

**Behavioral effects induced by organic insecticides can be exploited for a sustainable control of the Orange Spiny Whitefly Aleurocanthus spiniferus**

Selma Mokrane1, Giuseppe Cavallo1, Francesco Tortorici2, Elena Romero3, Alberto Fereres3, Khaled Djelouah1, Vincenzo Verrastro1, Daniele Cornara1,3*

1 International Centre for Advanced Mediterranean Agronomic Studies - Institute of Bari (CIHEAM-Bari). Via Ceglie 9, 70010 Valenzano (BA), Italy

2 Dipartimento di Scienze Agrarie, Forestali ed Alimentari DISAFA, University of Torino, Largo Braccini 2, 10095 Grugliasco (TO), Italy

3 Instituto de Ciencias Agrarias (ICA). Consejo Superior de Investigaciones Científicas (CSIC). Calle Serrano 115dpdo, 28006 Madrid, Spain

* Corresponding author: [danielecornara@gmail.com](mailto:danielecornara@gmail.com)

**Table S1** Efficacy of the products (lethal toxicity) on the different OSW instars: Negative Binomial generalized linear model followed by Tukey test for pairwise comparison (contrast). Model baselines: azadirachtin

**First Instar nymphs**

Estimate Std. Error z value Pr(>|z|)

(Intercept) 4.2547767 0.1306557 32.565 <2e-16 ***

CT -0.0627775 0.1753829 -0.358 0.7204

EO -0.0085388 0.1728746 -0.049 0.9606

Mineral oil 0.1341019 0.1752053 0.765 0.4440

Pyrethrin -0.0969278 0.1812929 -0.535 0.5929

Untreated -0.3085021 0.1815360 -1.699 0.0892 .

time 0.0023857 0.0029790 0.801 0.4232

CT:time -0.0034586 0.0040014 -0.864 0.3874

EO:time -0.0034481 0.0039440 -0.874 0.3820

Mineral oil :time -0.0044825 0.0039979 -1.121 0.2622

Pyrethrin:time 0.0001541 0.0041332 0.037 0.9703

Untreated:time 0.0030456 0.0041358 0.736 0.4615

contrast estimate SE df z.ratio p.value

Azadirachtin - CT 0.17345 0.120 Inf 1.448 0.6974

Azadirachtin - EO 0.11888 0.118 Inf 1.007 0.9159

Azadirachtin - Mineral oil 0.00934 0.120 Inf 0.078 1.0000

Azadirachtin - Pyrethrin 0.09200 0.124 Inf 0.743 0.9765

Azadirachtin - Untreated 0.21104 0.124 Inf 1.704 0.5292

CT - EO -0.05457 0.111 Inf -0.491 0.9965

CT - Mineral oil -0.16412 0.113 Inf -1.453 0.6943

CT - Pyrethrin -0.08146 0.117 Inf -0.695 0.9826

CT - Untreated 0.03759 0.117 Inf 0.320 0.9996

EO - Mineral oil -0.10954 0.111 Inf -0.986 0.9227

EO - Pyrethrin -0.02688 0.116 Inf -0.233 0.9999

EO - Untreated 0.09216 0.116 Inf 0.797 0.9681

Mineral oil - Pyrethrin 0.08266 0.117 Inf 0.706 0.9813

Mineral oil - Untreated 0.20170 0.117 Inf 1.720 0.5184

Pyrethrin - Untreated 0.11905 0.121 Inf 0.980 0.9243

**Second and Third Instar nymphs**

Estimate Std. Error z value Pr(>|z|)

(Intercept) 4.1988098 0.1290281 32.542 <2e-16 ***

CT 0.0227050 0.1730683 0.131 0.896

EO -0.0321969 0.1707226 -0.189 0.850

Mineral oil 0.1139144 0.1730168 0.658 0.510

Pyrethrin 0.0043122 0.1789143 0.024 0.981

Untreated -0.0640270 0.1790213 -0.358 0.721

time -0.0044592 0.0029521 -1.511 0.131

CT:time 0.0017961 0.0039577 0.454 0.650

EO:time -0.0001071 0.0039063 -0.027 0.978

Mineral oil :time -0.0024684 0.0039609 -0.623 0.533

Pyrethrin:time 0.0009472 0.0040922 0.231 0.817

Untreated:time -0.0010184 0.0040977 -0.249 0.804

contrast estimate SE df z.ratio p.value

Azadirachtin - CT -0.080179 0.118 Inf -0.677 0.9845

Azadirachtin - EO 0.035625 0.117 Inf 0.305 0.9996

Azadirachtin - Mineral oil -0.034924 0.118 Inf -0.295 0.9997

Azadirachtin - Pyrethrin -0.034624 0.122 Inf -0.283 0.9998

Azadirachtin - Untreated 0.096617 0.123 Inf 0.788 0.9696

CT - EO 0.115804 0.110 Inf 1.054 0.8994

CT - Mineral oil 0.045254 0.112 Inf 0.406 0.9986

CT - Pyrethrin 0.045555 0.116 Inf 0.393 0.9988

CT - Untreated 0.176796 0.116 Inf 1.525 0.6480

EO - Mineral oil -0.070549 0.110 Inf -0.642 0.9878

EO - Pyrethrin -0.070248 0.114 Inf -0.615 0.9900

EO - Untreated 0.060992 0.114 Inf 0.533 0.9948

Mineral oil - Pyrethrin 0.000301 0.116 Inf 0.003 1.0000

Mineral oil - Untreated 0.131541 0.116 Inf 1.134 0.8672

Pyrethrin - Untreated 0.131240 0.120 Inf 1.093 0.8842

**Fourth instar nymphs (puparia)**

Estimate Std. Error z value Pr(>|z|)

(Intercept) 3.8417746 0.1456455 26.378 <2e-16 ***

CT -0.3158457 0.1958732 -1.613 0.107

EO -0.2985951 0.1931283 -1.546 0.122

Mineral oil -0.1588065 0.1956394 -0.812 0.417

Pyrethrin -0.3117112 0.2024570 -1.540 0.124

Untreated -0.2937430 0.2024748 -1.451 0.147

time -0.0026538 0.0033291 -0.797 0.425

CT:time 0.0017007 0.0044755 0.380 0.704

EO:time -0.0010157 0.0044172 -0.230 0.818

Mineral oil :time -0.0003932 0.0044729 -0.088 0.930

Pyrethrin:time 0.0036909 0.0046226 0.798 0.425

Untreated:time 0.0004647 0.0046283 0.100 0.920

contrast estimate SE df z.ratio p.value

Azadirachtin - CT 0.2614 0.134 Inf 1.952 0.3702

Azadirachtin - EO 0.3311 0.132 Inf 2.506 0.1222

Azadirachtin - Mineral oil 0.1714 0.134 Inf 1.281 0.7957

Azadirachtin - Pyrethrin 0.1936 0.138 Inf 1.399 0.7275

Azadirachtin - Untreated 0.2789 0.138 Inf 2.014 0.3342

CT - EO 0.0697 0.125 Inf 0.559 0.9936

CT - Mineral oil -0.0900 0.126 Inf -0.712 0.9806

CT - Pyrethrin -0.0678 0.131 Inf -0.517 0.9955

CT - Untreated 0.0174 0.131 Inf 0.133 1.0000

EO - Mineral oil -0.1597 0.125 Inf -1.282 0.7951

EO - Pyrethrin -0.1375 0.129 Inf -1.062 0.8963

EO - Untreated -0.0522 0.130 Inf -0.403 0.9986

Mineral oil - Pyrethrin 0.0222 0.131 Inf 0.169 1.0000

Mineral oil - Untreated 0.1075 0.131 Inf 0.819 0.9642

Pyrethrin - Untreated 0.0853 0.136 Inf 0.627 0.9890

**Adults**

Estimate Std. Error z value Pr(>|z|)

(Intercept) 1.6365029 0.1968751 8.312 < 2e-16 ***

CT -1.0192209 0.2765182 -3.686 0.000228 ***

EO -0.6934721 0.2667089 -2.600 0.009319 **

Mineral oil -0.7946150 0.2730271 -2.910 0.003610 **

Pyrethrin -0.5746732 0.2791306 -2.059 0.039514 *

Untreated -0.7579627 0.2811164 -2.696 0.007012 **

time -0.0025286 0.0044950 -0.563 0.573745

CT:time 0.0073946 0.0062361 1.186 0.235710

EO:time 0.0036917 0.0060736 0.608 0.543304

Mineral oil :time 0.0004773 0.0062658 0.076 0.939280

Pyrethrin:time -0.0023519 0.0064520 -0.365 0.715466

Untreated:time 0.0069269 0.0063560 1.090 0.275789

contrast estimate SE df z.ratio p.value

Azadirachtin - CT 0.78212 0.187 Inf 4.182 0.0004

Azadirachtin - EO 0.57510 0.182 Inf 3.167 0.0192

Azadirachtin - Mineral oil 0.77931 0.187 Inf 4.170 0.0004

Azadirachtin - Pyrethrin 0.65008 0.192 Inf 3.386 0.0093

Azadirachtin - Untreated 0.53586 0.190 Inf 2.813 0.0554

CT - EO -0.20702 0.179 Inf -1.157 0.8571

CT - Mineral oil -0.00281 0.184 Inf -0.015 1.0000

CT - Pyrethrin -0.13204 0.189 Inf -0.697 0.9823

CT - Untreated -0.24626 0.188 Inf -1.310 0.7795

EO - Mineral oil 0.20421 0.179 Inf 1.142 0.8636

EO - Pyrethrin 0.07498 0.184 Inf 0.407 0.9986

EO - Untreated -0.03924 0.183 Inf -0.215 0.9999

Mineral oil - Pyrethrin -0.12923 0.189 Inf -0.683 0.9839

Mineral oil - Untreated -0.24345 0.188 Inf -1.296 0.7872

Pyrethrin - Untreated -0.11422 0.193 Inf -0.592 0.9916

**Table S2** Effect of the insecticides on eggs hatching, i.e. number of first instar nymphs emerged/alive upon emergence over the total number of eggs laid and exposed to the treatment: raw data

| **Treatment** | **n leaves** | **n eggs** | **n emerged nymphs** | **Emergence rate (%)** |
| --- | --- | --- | --- | --- |
| **EO** | 47 | 747 | 273 | 36.54 |
| **CT** | 43 | 1571 | 508 | 32.33 |
| **Mineral oil** | 45 | 931 | 515 | 55.31 |
| **Azadirachtin** | 46 | 974 | 540 | 55.44 |
| **Pyrethrin** | 50 | 756 | 82 | 10.84 |
| **Untreated** | 26 | 463 | 119 | 25.70 |

**Table S3** Effect of the insecticides on eggs hatching, i.e. number of first instar nymphs emerged/alive upon emergence over the total number of eggs laid and exposed to the treatment: Kruskal-Wallis followed by Wilcoxon rank sum test for pairwise comparisons

**Kruskal-Wallis rank sum test**

Kruskal-Wallis chi-squared = 30.474, df = 5, p-value = 1.19e-05

**Pairwise comparisons using Wilcoxon rank sum test**

data: y and egg$treatment

Azadirachtin CT EO Mineral oil Pyrethrin

CT 0.29186 - - - -

EO 0.07001 0.63618 - - -

Mineral oil 0.13322 0.84429 0.67381 - -

Pyrethrin 5.5e-06 0.00201 0.00991 0.00042 -

Untreated 0.04895 0.40983 0.67682 0.40983 0.07001

**Table S4** Effect of the insecticides on OSW adults host searching behavior, i.e. on the number of adults alighting on citrus plants: scheme of the cage (positions)

| **West** | **North** | | | **East** |
| --- | --- | --- | --- | --- |
|  | A | B | C |  |
|  | D | E | F |  |
|  | **South** | | |  |

**Table S5** Effect of the insecticides on OSW adults host searching behavior, i.e. on the number of adults alighting on citrus plants: Kruskal-Wallis followed by Wilcoxon rank sum test for pairwise comparisons

**Kruskal-Wallis rank sum test**

Kruskal-Wallis chi-squared = 83.879, df = 5, p-value < 2.2e-16

**Pairwise comparisons using Wilcoxon rank sum test**

Azadirachtin CT EO Mineral oil Pyrethrin

CT 0.01207 - - - -

EO 0.03630 6.2e-06 - - -

Mineral oil 7.2e-08 0.00422 1.8e-13 - -

Pyrethrin 0.45203 0.00127 0.17409 9.1e-10 -

Untreated 0.06679 0.45203 0.00011 0.00030 0.01044

**Table S6** Effect of the insecticides on OSW oviposition, i.e. on the number of plants on which OSW laid eggs. Kruskal-Wallis followed by Wilcoxon rank sum test for pairwise comparisons

**Kruskal-Wallis rank sum test**

Kruskal-Wallis chi-squared = 20.796, df = 5, p-value = 0.0008851

**Pairwise comparisons using Wilcoxon rank sum test**

Azadirachtin CT EO Mineral oil Pyrethrin

CT 0.285 - - - -

EO 0.088 0.407 - - -

Mineral oil 0.160 0.655 0.607 - -

Pyrethrin 0.034 0.160 0.407 0.265 -

Untreated 0.504 0.103 0.034 0.058 0.015
